# Evaluation of Two Equations for Prediction of Digestible Energy in Mixed Feeds and Diets for Horses

**DOI:** 10.3390/ani12131628

**Published:** 2022-06-24

**Authors:** Andrés Luis Martínez Marín, Emanuela Valle, Domenico Bergero, Francisco Requena, Claudio Forte, Achille Schiavone

**Affiliations:** 1 Dipartimento di Scienze Veterinarie, University of Torino, Largo Paolo Braccini 2, 10095 Grugliasco, Italy; pa1martm@uco.es (A.L.M.M.); emanuela.valle@unito.it (E.V.); domenico.bergero@unito.it (D.B.); claudio.forte@unito.it (C.F.); achille.schiavone@unito.it (A.S.); 2 Departamento de Biología Celular, Fisiología e Inmunología, Universidad de Córdoba, Ctra. Madrid-Cádiz km 396, 14071 Córdoba, Spain

**Keywords:** digestibility, energy, feeding, nutrition, equine

## Abstract

**Simple Summary:**

Horses need energy from feeds consumed to maintain health and performance. Digestible energy is a common form to express the energy value of feeds for horses. Measurement of digestible energy in feeds requires animal assays that are difficult to conduct. Thus, several researchers have developed empirical equations to predict digestible energy in horse feeds from their chemical composition. In the present study, we evaluated two of those equations that predict digestible energy from the chemical composition declared on the label of commercial mixed feeds and diets in Europe. After testing both equations against 32 mixed feeds and diets of known digestible energy content, we found that one performed slightly better than the other although both provided acceptable predictions. Our results suggest that the effects of crude fiber on the digestibility of the other proximate components should be reexamined in both equations.

**Abstract:**

Several authors have developed equations for estimating digestible energy in horse feeds as an alternative to the inconveniences of in vivo digestibility assays. We aimed to evaluate two of such equations. A dataset was constructed from the literature with 32 mixed feeds and diets of known proximate composition, whose digestibility was measured in in vivo assays. Then, the digestible energy of the mixed feeds and diets was predicted with both equations from their proximate components. Precision, accuracy, reproducibility, bias, and decomposition of total error of predictions were determined. Both equations performed almost equally well (R^2^ = 0.89 vs. 0.87, root mean square error of prediction = 183 vs. 217 kcal/kg dry matter, concordance correlation coefficient = 0.91 vs. 0.86, and linear error = 24.6 vs. 33.6% of total error). Linear bias (*p* < 0.01 in both equations) resulted in overvaluation of low digestible energy feeds and, to a lesser extent, undervaluation of high digestible energy feeds and was significantly (*p* < 0.05) related to crude fiber. The obtained results indicate that the accuracy of both equations could be improved by reassessing the effects of crude fiber on the digestibility of the other proximate components.

## 1. Introduction

Balanced diets for horses should provide energy and nutrients required for body maintenance, growth, reproduction, and work, as well as help prevent metabolic problems [1]. Energy is not a nutrient, but a property of the so-called “energy-yielding” nutrients (carbohydrates, protein, and fat) contained in the diet. An accurate estimation of the energy provided by the diet is necessary for optimal production and performance [2]. Energy content in animal feeds can be described in terms of gross energy, digestible energy, metabolizable energy, or net energy. Gross energy accounts for the heat of combustion or total heat yielded from complete oxidation of the feed in bomb calorimetry. Although it is a very precise measure, it is not a satisfactory descriptor of the energy value for the animal because it only depends on the chemical composition and does not consider that nutrients are not equally digested nor used in the metabolism. Digestible, metabolizable, and net energy terms account for the energy that is available to the animal after the successive losses that occur in feces after digestion of feed components, as fermentation gases in the digestive tract and nitrogen compounds in urine, and when absorbed nutrients are used to fuel the physiological functions, respectively [3]. Energy lost in feces is the largest and most variable of the energy losses among feeds, hence determining digestible energy is a basic step to establish the metabolizable and net energy contents of horse feeds [4,5].

In vivo digestibility studies are the gold standard for measuring digestible energy in horse feeds, but because those assays are time consuming, labor intensive, costly, and often highly impractical, researchers have made several efforts to develop predictive equations based on the chemical composition of the feeds [6,7,8,9,10,11,12,13,14]. Some of those equations have been incorporated into the models proposed by several feed evaluation systems for horses [15,16,17]. Digestible energy of feedstuffs is predicted in the NRC system [17] from the contents of various chemical components with specialized equations for forages, concentrates, and fat and oils proposed by several researchers [6,9,14]. The INRA system [16] proposes a unique equation to predict the digestibility coefficient of gross energy from the digestibility of the organic matter, using a specific correction dedicated to concentrates or forages based on experimental digestion data; then, digestible energy is calculated by multiplying the gross energy by its digestibility coefficient [7]. In the GfE system [15], a unique equation using the contents of proximate components and their experimental digestion coefficients is used for predicting the digestible energy [14]. Recently, a new unique model was proposed by Núñez-Sánchez et al. [8]. This model is designed and calculated using a similar approach to the GfE model [15].

Some of the proposed predictive equations are not of practical use in Europe for rapid assessment of digestible energy in commercial mixed feeds (compound feeds) and mixed diets (forage plus concentrates) because they do not rely on the proximate components [6,9,11,12,13], including crude fiber [18], that are compulsory on the label [19], but on Van Soest’s analytical method [20]. Furthermore, when using a prediction equation, it is important both to know the range of nutrient composition where it is applicable and to bear in mind that the summative equations based on the digestibility of individual nutrients are usually more robust than those obtained by regression methods because the estimate of the digestibility of the nutrients used for the summative equations can be secured by testing the nutritional uniformity of the linear relationship between each nutrient and its digestibility, meaning the physiological background [4].

Zeyner and Kienzle [14] developed an equation to estimate the digestible energy of mixed feeds and diets for horses from their proximate components by combining data from 170 digestion trials and testing it using an additional set of observations not included in the development dataset [12,13]. The authors did not offer many details of both datasets in the published paper. The original equation was slightly modified later because of minorly changed assumptions on the gross energy content of crude nutrients from diets of large herbivores recommended by the German Society of Nutrition Physiology [4]. Núñez-Sánchez et al. [8] presented an equation for the prediction of the digestible energy in ingredients for horses from their proximate composition that was modeled from the results of a total of 16 published papers. When applied to a set of 116 feedstuffs of known composition, the equation showed a very good agreement with the modified equation of Zeyner and Kienzle [14], adopted by GfE [15], and those of Fonnesbeck [6] and Pagan [9] used by NRC [17] to predict the digestible energy of concentrates and forages, respectively. However, no evaluation with in vivo observed values was carried out by Núñez-Sánchez et al. [8].

The current study was conducted to evaluate the equation of Núñez-Sánchez et al. [8] and the modified equation of Zeyner and Kienzle [14], as presented in Kienzle and Zeyner [4], to predict the digestible energy of mixed feeds and diets for horses, by using data collected from in vivo digestibility trials.

## 2. Materials and Methods

### 2.1. Creation of the Dataset for the Evaluation

A dataset with proximate composition and digestibility values of mixed feeds and diets that were obtained in in vivo digestibility trials was created from two sources. First, the digestible energy of 18 mixed diets assayed by Lindsey et al. [21] in a total of 40 individual measurements was calculated by multiplying the proximate components of the diets by their observed digestibility coefficients and their gross energy values (kcal/g: protein, 5.71; fat, 9.51; crude fiber, 4.80, and nitrogen-free extract, 4.18 [4]) (Table 1). Most diets comprised grass hay (mostly timothy) and one or two non-forage ingredients. Second, the keywords “digestibility” and “horses” were searched in Google Scholar. All the studies included in the work of Núñez-Sánchez et al. [8], as well as those papers that did not report the proximate composition or did not offer enough information to derive it from feed composition tables, were discarded. Five papers published between 1966 and 2020 that assayed 14 mixed feeds or diets in a total of 52 horses were retained [22,23,24,25,26]. Digestible energy in those papers was calculated by multiplying the proximate components by their observed digestibility coefficients and their gross energy values [22,25], or multiplying the gross energy by its digestibility coefficient, either calculated from the organic matter digestibility [23,26], as proposed by Martin-Rosset et al. [7], or directly reported in the paper [24] (Table 1). Next, digestible energy content in the 32 mixed feeds and diets was predicted from their proximate composition (Table 1), according to the equation of Núñez-Sánchez et al. [8] and the modified equation of Zeyner and Kienzle [14], as presented in Kienzle and Zeyner [4] (Equations (1) and (2), respectively).

Digestible energy (kcal/kg dry matter) = –236 + 48.5 × CP – 6.7 × CF + 37.3 × NFE + 90.1 × EE(1)

Digestible energy (kcal/kg dry matter) = –846 + 50.0 × CP + 2.4 × CF + 44.2 × NFE + 100.4 × EE (2)

CP, CF, NFE, and EE stand for crude protein, crude fiber, nitrogen-free extract, and crude fat, respectively, expressed as percentages on a dry matter basis.

### 2.2. Statistical Analysis

Statistical analysis was performed with SAS OnDemand for Academics (SAS Institute, Cary, NC, USA). Precision, accuracy, and reproducibility of Equations (1) and (2) were established from the coefficient of determination (R^2^) obtained by linear regression analysis, the root mean square error of prediction (RMSEP) calculated from the residuals, and the concordance correlation coefficient (CCC) [27], respectively. Existence of mean bias and linear bias was determined by regression of the residuals on the predicted values centered in the predicted mean [28]. Moreover, the mean square error of prediction (MSEP) was decomposed into central tendency error, linear error, and random error [29]. Statistical significance was declared at *p* < 0.05.

## 3. Results and Discussion

### 3.1. Characteristics of the Dataset for the Evaluation

The dataset included 24 mixed diets and eight mixed feeds (Table 1). Except for one mixed feed in the study of Smolders et al. [26] and the mixed diet of Parkins et al. [24], which were of unknown composition, the other 30 mixed feeds and diets used in the experimental treatments included a total of 35 different ingredients. Grass hay, oats, wheat bran, and maize were the most utilized ingredients (19, 10, 10, and 9 experimental treatments, respectively). Twelve ingredients were only included in one of the 30 experimental treatments (maize bran, maize cobs, oat feed, oat hulls, faba beans, rice bran, soybean meal, grass meal, wheat, palm kernel expeller, lupin seeds, and molassed sugar beet pulp). The proximate composition of the mixed feeds and diets showed large variability: 6.3–21.6% crude protein, 3.5–31.9% crude fiber, 50.4–75.5% nitrogen-free extract, and 2.0–5.3% crude fat (Table 1). That composition was within the ranges in the study of Zeyner and Kienzle [14], except for the nitrogen-free extract (5.7–28.7% crude protein, 4.2–34.7% crude fiber, and 33.8–69.8% nitrogen-free extract), and in the study of Núñez-Sánchez et al. [8] (2.7–70.3% crude protein, 0.0–53.3% crude fiber, 14.2–89.1% nitrogen-free extract, and 0.1–44.7% crude fat). Observed digestible energy ranged from 2016 to 3531 kcal/kg dry matter, with a mean of 2599 ± 492 kcal/kg dry matter. Thus, the mixed feeds and diets used for the validation covered a broad range of digestible energy values.

Observed digestible energy was positively correlated with crude protein, nitrogen-free extract, and crude fat (r = 0.83, 0.53 and 0.48, respectively, *p* < 0.01), but negatively correlated with crude fiber (r = −0.94, *p* < 0.001). In this regard, in a study with ruminants involving 106 feedstuffs, it was found that crude fiber not only hardly contributes to energy value despite its moderate digestibility but would also decrease digestion of other nutrients or increase endogenous fecal losses of energy, thus reducing digestible energy [30]. Again, it could be calculated that within the range of nutrient composition in the dataset each gram of digestible organic matter supplied on average 4.60 ± 0.11 kcal of digestible energy, which is very close to the 4.54 ± 0.49 kcal that can be calculated from the results of several studies with forages and mixed diets [6,11,31,32,33].

Results from in vivo assays with horses have demonstrated a close and linear relationship between energy digestibility and organic matter digestibility in forages, concentrates, and mixed diets [7,10,11,34]. In the present work, energy digestibility of the mixed feeds and diets in Table 1, derived from their calculated gross energy and digestible energy predicted with Equations (1) and (2), was highly correlated (r > 0.91; *p* < 0.001) with the observed organic matter digestibility in the dataset, which would support the reliability of summative equations based on digestible nutrients to predict digestible energy of mixed feeds and diets for horses [4].

### 3.2. Performance of the Equations

Equations (1) and (2) have the same structure. Both are summative equations that result from adding the equations for the prediction of the digestible energy supplied by each digestible nutrient into a single equation [35]. An important difference between Equations (1) and (2) is that crude fiber has a negative contribution to digestible energy in the former but a positive contribution in the latter. This difference is due to the fact that the crude fiber term derives from the equation for the calculation of digestible energy provided by crude fiber (positive contribution) and from the equation for the calculation of digestible energy provided by nitrogen-free extract (negative effect of a higher magnitude of crude fiber) in Equation (1) [8].

**Figure 1:**
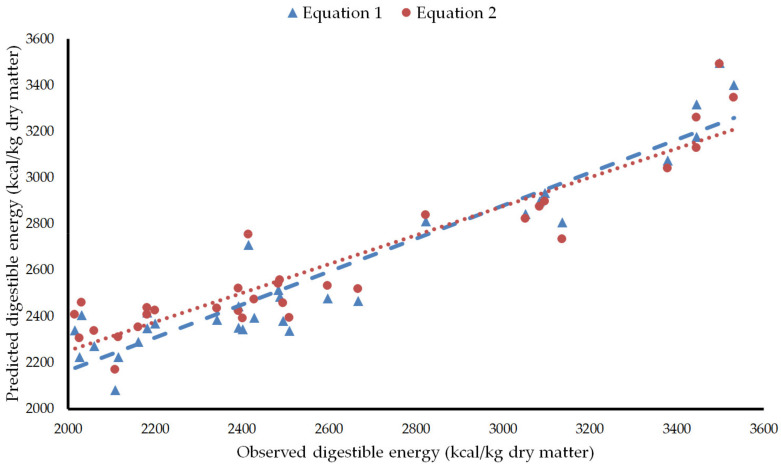
Plot of predicted versus measured values of digestible energy. Equation 1: Equation of Núñez-Sánchez et al. [8]. Equation 2: Equation of Zeyner and Kienzle [14] modified as presented in Kienzle and Zeyner [4].

**Figure 2:**
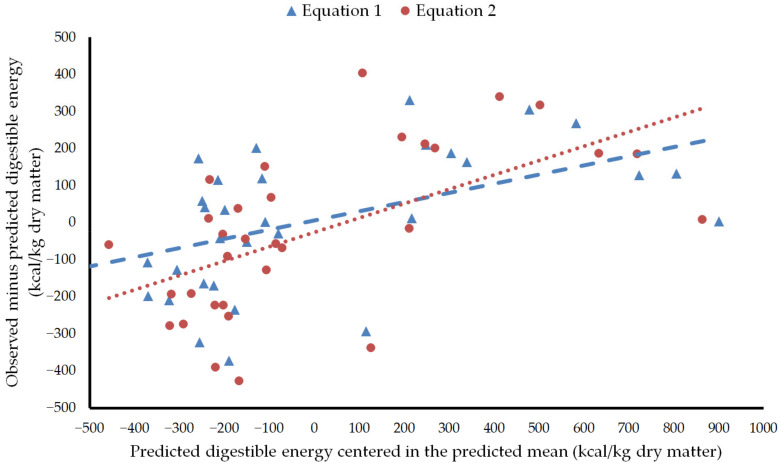
Plot of residuals versus predicted digestible energy centered in the predicted mean. Equation 1: Equation of Núñez-Sánchez et al. [8]. Equation 2: Equation of Zeyner and Kienzle [14] modified as presented in Kienzle and Zeyner [4].

Evaluation showed that Equation (1) had high precision (R^2^ = 0.89), accuracy (RMSEP = 183 kcal/kg dry matter or 7.1% of the observed mean), and reproducibility (CCC = 0.91) (Figure 1). Equation (2) performed slightly worst (R^2^ = 0.87, RMSEP = 217 kcal/kg dry matter or 8.4% of the observed mean, and CCC = 0.86) (Figure 1). The regression of the residuals on the predicted digestible energy values centered in the predicted mean showed that no equation had mean bias (intercept *p* > 0.05), whereas linear bias was significant in both of them (slope *p* < 0.01) (Figure 2). Central tendency error, linear error, and random error accounted for 0.09, 24.56, and 75.35% of MSEP, respectively, in Equation (1), while they represented 1.52, 33.63, and 64.86% of MSEP, respectively, in Equation (2). Both equations overvalued mixed feeds and diets with low digestible energy and undervalued mixed feeds and diets with high digestible energy, although the error was higher with Equation (2). In the six mixed feeds and diets with observed digestible energy beyond one standard deviation under the mean, overprediction was 9.7 ± 6.5% and 13.3 ± 6.1% with Equations (1) and (2), respectively. In the eight mixed feeds and diets with observed digestible energy beyond one standard deviation over the mean, underprediction was 6.2 ± 3.5% and 7.7 ± 4.1% with Equations (1) and (2), respectively. Zeyner and Kienzle [14] recognized that their equation would underestimate digestible energy in diets with more than 5% fat or rich in highly fermentable fiber. However, only one mixed feed beyond one standard deviation over the mean had more than 5% fat (No. 27 in Table 1) or would be expected to contain a high proportion of highly fermentable fiber (No. 27 and 28 in Table 1, i.e., Compound feeds 21 and 23 of Smolders et al. [26]).

Taking Equation (1) as an example, the prediction error when feeding a horse of 500 kg body weight undergoing light exercise would translate into 1 kg weight loss every 10 days if fed a low energy diet, or 1 kg weight gain every 16 days if fed a high energy diet [17]. Such errors could deviate body weight and body condition score from ideal values and negatively affect horse health and performance in the medium term [36], and should be taken into account in specialized equine nutrition counseling [37]. Nevertheless, it is worth mentioning that in the 18 mixed feeds and diets whose digestible energy was within one standard deviation of the mean (which corresponded to a crude fiber content between 16.5 and 28.5% dry matter), the mean error of estimation would be reduced to 4.77 ± 3.51% and 5.30 ± 4.06% with Equations (1) and (2), respectively, or approximately ±120 kcal/kg dry matter.

**Figure 3:**
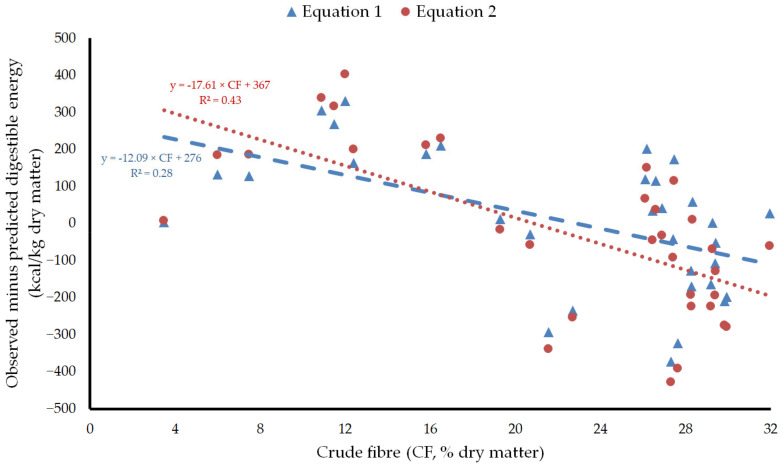
Plot of residuals versus crude fiber content in feeds. Equation 1: Equation of Núñez-Sánchez et al. [8]. Equation 2: Equation of Zeyner and Kienzle [14] modified, as presented in Kienzle and Zeyner [4].

Due to the close relationship between gross energy digestibility, organic matter digestibility, and crude fiber in horse feeds [7,38,39], crude fiber might be responsible for the linear bias in the predictions of digestible energy by both equations. This would be supported by the fact that the residuals were negatively correlated with crude fiber (r = −0.53 and *p* < 0.01 in Equation (1), and r = −0.66 and *p* < 0.001 in Equation (2); Figure 3), i.e., the lower the crude fiber content in the feed, the higher the underestimation of its digestible energy value, and the higher the crude fiber content in the feed the higher the overestimation of its digestible energy value. The equations used by Núñez-Sánchez et al. [8] to derive digestible contents of crude protein and crude fat in ingredients did not take into account any possible negative effect of crude fiber on the digestion of those proximate components. Zeyner and Kienzle [14] arbitrarily set the contribution of digestible crude fiber to digestible energy to a value 5.33 times lower than expected from its heat of combustion to account for any negative effects of crude fiber on the digestion of the other proximate components. Owens et al. [30] showed that crude fiber increases the fecal losses of crude protein, crude fat, and nitrogen-free extract and thus decreases the digestible energy value of feedstuffs in ruminants. The same might be true in horses. The high and negative correlations found in the dataset used in the present work (Table 1) between crude fiber and digestible crude protein, digestible nitrogen-free extract, and digestible crude fat (r = −0.91, −0.83, and −0.87, respectively; *p <* 0.001) would indicate that any negative effects of crude fiber on the digestibility of each of the other proximate components, as done only for nitrogen-free extract in Núñez-Sánchez et al. [8], should be considered in Equations (1) and (2) in order to improve their accuracy.

The equations evaluated in the present work assume that digestible energy values of feedstuffs are additive in the mixed diets where they are included and do not take into account any digestive interactions that may occur between them, which is a well-known limitation [40]. Those interactions, or associative effects either negative or positive, are not always observed [23,41], but could exist in mixed diets with high levels of starch-rich concentrates or based on low-quality forages [31,40]. Moreover, other factors that could affect the accuracy of predicted digestible energy with those equations are the technological treatment of the concentrates [42,43,44,45] and the particle size of forages [46,47,48].

## 4. Conclusions

Two summative equations developed for the prediction of digestible energy in horse feedstuffs (Equation (1)) or mixed feeds (Equation (2)) from their proximate composition were evaluated against a dataset of 32 mixed feeds and diets whose digestible energy was derived from the results of in vivo digestibility trials. Under the conditions examined, both equations showed good precision and accuracy, but Equation (1) performed slightly better than Equation (2). Due to linear bias, both equations overestimated and, to a lesser extent, underestimated mixed feeds and diets with low and high digestible energy, respectively, which could be due to an inaccurate assessment of the effects of crude fiber on the digestibility of the other proximate components. Under practical feeding conditions, digestible energy of commercial mixed feeds and diets for horses can be predicted from proximate components declared on the label with satisfactory precision and accuracy by both equations when crude fiber content ranges from 16.5 to 28.5% dry matter. However, feeding plans based on predicted digestible energy of commercial mixed feeds and diets whose crude fiber content is outside the above range might negatively affect the body condition and performance of horses in the medium term.

## Figures and Tables

**Table 1:** Crude nutrient composition (%), digestible nutrient composition (%) and observed and predicted digestible energy content (kcal/kg) of the mixed feeds and diets included in the evaluation. All values on dry matter basis.

No. ^1^	Crude Nutrients ^2^				Digestible Nutrients ^3^				DE ^4^		
	CP	CF	NFE	EE	DCP	DCF	DNFE	DEE	Obs	Pred_NS_	Pred_ZK_
1	13.7	19.3	58.8	3.6	10.6	2.5	45.8	1.9	2823	2811	2840
2	11.9	21.6	58.2	3.8	7.8	6.2	37.0	1.3	2415	2709	2754
3	7.8	26.9	58.4	2.3	4.4	10.5	38.7	0.2	2392	2351	2424
4	11.9	29.2	50.9	3.4	7.5	11.8	26.5	0.8	2183	2348	2407
5	7.2	26.5	59.4	2.7	4.5	0.0	50.7	0.6	2428	2395	2474
6	7.3	26.6	59.3	2.5	5.3	0.0	50.8	0.7	2495	2380	2458
7	6.7	29.4	57.6	2.0	3.0	12.8	31.0	0.4	2116	2223	2309
8	14.8	29.3	53.5	2.3	9.6	13.1	30.8	0.2	2487	2486	2556
9	13.3	29.4	54.8	2.1	7.9	12.8	31.8	0.0	2392	2444	2521
10	12.1	28.3	50.4	3.4	8.2	12.1	30.0	1.1	2402	2344	2393
11	11.8	28.3	51.5	3.4	8.2	10.6	26.2	1.4	2200	2370	2425
12	11.7	27.4	52.2	3.2	8.2	11.0	30.7	0.6	2342	2385	2435
13	11.3	27.3	53.2	3.2	7.4	8.9	27.0	0.6	2032	2405	2460
14	7.5	29.9	55.0	2.7	4.4	11.5	28.1	0.5	2027	2225	2306
15	6.3	31.9	54.2	2.3	2.5	14.6	29.1	0.5	2109	2082	2170
16	10.4	29.8	51.8	3.0	6.5	11.2	25.5	0.9	2061	2271	2336
17	8.7	28.2	54.4	2.9	5.1	9.8	32.5	0.5	2161	2289	2354
18	8.6	27.6	55.5	3.0	4.8	10.4	28.5	0,5	2016	2339	2408
19	17.7	15.8	54.2	4.0	13.3	5.3	43.1	2.8	3085	2899	2799
20	17.0	16.5	54.7	3.6	12.9	5.8	43.3	2.3	3052	2843	2752
21	13.6	3.5	75.5	3.1	Not available				3499	3496	3429
22	21.6	6.0	64.2	2.6					3531	3400	3285
23	11.3	22.7	55.7	2.0	8.1	5.4	32.3	1.4	2182	2418	2387
24	10.0	27.5	54.3	2.8	6.1	12.2	34.1	1.6	2509	2337	2342
25	11.0	26.1	53.0	4.2	7.1	11.5	32.8	2.9	2598	2479	2462
26	11.0	26.2	52.9	4.1	7.2	12.1	33.6	2.8	2667	2466	2450
27	20.6	11.5	54.0	5.3	Not available				3446	3178	3038
28	18.0	12.0	55.0	2.2					3137	2806	2673
29	16.7	10.9	59.8	3.8					3379	3074	2966
30	15.6	12.4	57.5	3.9					3097	2934	2821
31	9.2	20.7	59.2	2.6					2484	2514	2487
32	20.5	7.5	59.1	4.5					3446	3318	3177

^1^ 1–18: Mixed diets from Lindsey et al. [21]. 19–20: Mixed diets from Hintz and Loy [22]. 21–22: Mixed feeds from Martin-Rosset and Dulphy [23]. 23: Mixed diet from Parkins et al. [24]. 24–26: Mixed diets from Saastamoinen and Särkijärvi [25]. 27–32: Mixed feeds from Smolders et al. [26]. Digestibility values of the mixed feeds in Martin-Rosset and Dulphy [23] and Smolders et al. [26] were obtained by the difference method. ^2^ CP: crude protein. CF: crude fiber. EE: crude fat. NFE: nitrogen-free extract. ^3^ DCP: digestible crude protein. DCF: digestible crude fiber. DNFE: digestible nitrogen-free extract. DEE: Digestible crude fat. ^4^ DE: digestible energy, either observed (Obs) in each diet or predicted with Equation (1) (Pred_NS_) and Equation (2) (Pred_ZK_).

## Data Availability

The data presented in this study are available in the article.

## References

[1] Bergero D., Valle E. (2007). A multi-factorial approach to the nutritional requirements of sports horses: Critical analysis and some practical applications. Ital. J. Anim. Sci..

[2] Becvarova I., Pleasant R.S., Thatcher C.D. (2009). Clinical assessment of nutritional status and feeding programs in horses. Veter Clin. N. Am. Equine Pract..

[3] National Research Council (NRC) (1981). Nutritional Energetics of Domestic Animals and Glossary of Energy Terms.

[4] Kienzle E., Zeyner A. (2010). The development of a metabolizable energy system for horses. J. Anim. Physiol. Anim. Nutr..

[5] Vermorel M., Martin-Rosset W. (1997). Concepts, scientific bases, structure and validation of the French horse net energy system (UFC). Livest. Prod. Sci..

[6] Fonnesbeck P.V. (1981). Estimating digestible energy and TDN for horses with chemical analysis of feeds. J. Anim. Sci..

[7] Martin-Rosset W., Vermorel M., Doreau M., Tisserand J., Andrieu J. (1994). The French horse feed evaluation systems and recommended allowances for energy and protein. Livest. Prod. Sci..

[8] Núñez-Sánchez N., Requena-Domenech F., García-Moya M., Peña-Blanco P., Agüera-Buendía E., Martínez-Marín A.L. (2019). Predicción del contenido en energía digestible de alimentos para equinos a partir de la composición química proximal. Rev. Científica FVC-LUZ.

[9] Pagan J.D., Pagan J.D. (1998). Measuring the digestible energy content of horse feeds. Advances in Equine Nutrition.

[10] Sales J., Homolka P., Koukolová V. (2013). Prediction of energy digestibility of hays in horses. J. Equine Veter Sci..

[11] Williams M.J. (2020). Effects of Energy Source and Amount on Nutrient Digestibility and Prediction of Digestible Energy in Horses. Ph.D. Thesis.

[12] Zeyner A. (1995). Ermittlung des Gehaltes an verdaulicher Energie im Pferdefutter über die Verdaulichkeitsschätzung. Übers Tierernährg.

[13] Zeyner A., Hoffmann M., Fuchs R. (1992). Möglichkeiten der schätzung des energiegehalts in rationen zur sportpferdefütterung. Pferdeheilkunde.

[14] Zeyner A., Kienzle E. (2002). A method to estimate digestible energy in horse feed. J. Nutr..

[15] Gesellschaft für Ernährungsphysiologie. (GfE) (2014). Emphelungen zur Energie- und Närhstoffversorgung von Pferden.

[16] Institut National Recherche Agronomique (INRA) (2015). Equine Nutrition, INRA Nutrient Requirements, Recommended Allowances and Feed Tables.

[17] National Research Council. (NRC) (2007). Nutrient Requirements of Horses.

[18] Henneberg W., Stohmann F. (1859). Uber das erhaltungsfutter volljahrigen rindviehs. J. Landwirtsch..

[19] EC (2009). Regulation 767/2009 of the European Parliament and of the Council of 13 July 2009 on the placing on the market and use of feed. Off. J..

[20] Van Soest P.J., Robertson J.B., Lewis B.A. (1991). Methods for dietary fiber, neutral detergent fiber, and nonstarch polysaccharides in relation to animal nutrition. J. Dairy Sci..

[21] Lindsey J.B., Beals C.L., Archibald J.G. (1926). The digestibility and energy values of feeds for horses. J. Agric. Res..

[22] Hintz H.F., Loy R.G. (1966). Effects of pelleting on the nutritive value of horse rations. J. Anim. Sci..

[23] Martin-Rosset W., Dulphy J. (1987). Digestibility interactions between forages and concentrates in horses: Influence of feeding level-comparison with sheep. Livest. Prod. Sci..

[24] Parkins J., Snow D., Adams S. (1982). The apparent digestibility of ‘complete diet’ cubes given to thoroughbred horses and the use of chromic oxide as an inert faecal marker. Br. Veter J..

[25] Saastamoinen M., Särkijärvi S. (2020). Effect of linseed (*Linum usitatissimum*) groats-based mixed feed supplements on diet nutrient digestibility and blood parameters of horses. Animals.

[26] Smolders E., Steg A., Hindle V. (1990). Organic matter digestibility in horses and its prediction. Neth. J. Agric. Sci..

[27] Lin L.I.-K. (1989). A concordance correlation coefficient to evaluate reproducibility. Biometrics.

[28] St-Pierre N. (2003). Reassessment of biases in predicted nitrogen flows to the duodenum by NRC 2001. J. Dairy Sci..

[29] Tedeschi L.O. (2006). Assessment of the adequacy of mathematical models. Agric. Syst..

[30] Owens F.N., Sapienza D.A., Hassen A.T. (2010). Effect of nutrient composition of feeds on digestibility of organic matter by cattle: A review1. J. Anim. Sci..

[31] Karlsson C.P., Lindberg J., Rundgren M. (2000). Associative effects on total tract digestibility in horses fed different ratios of grass hay and whole oats. Livest. Prod. Sci..

[32] Ragnarsson S., Lindberg J.E. (2010). Nutritional value of mixed grass haylage in Icelandic horses. Livest. Sci..

[33] Vermorel M., Martin-Rosset W., Vernet J. (1997). Energy utilization of twelve forages or mixed diets for maintenance by sport horses. Livest. Prod. Sci..

[34] Jensen R., Austbø D., Knudsen K.B., Tauson A.-H. (2014). The effect of dietary carbohydrate composition on apparent total tract digestibility, feed mean retention time, nitrogen and water balance in horses. Animal.

[35] Weiss W., Conrad H., Pierre N.S. (1992). A theoretically-based model for predicting total digestible nutrient values of forages and concentrates. Anim. Feed Sci. Technol..

[36] Harris P., Lindner A. (2005). Feeding the endurance horse. Applied Equine Nutrition.

[37] Vergnano D., Bergero D., Valle E. (2017). Clinical nutrition counselling service in the veterinary hospital: Retrospective analysis of equine patients and nutritional considerations. J. Anim. Physiol. Anim. Nutr..

[38] Almeida M.I.V.D., Ferreira W.M., Almeida F.Q.D., Gonçalves L.C., Rezende A.S.C. (1999). Composição química e predição do valor nutritivo de dietas para eqüinos. Rev. Bras. Zootec..

[39] Hallsworth E.G. (1949). The relationship between the crude-fibre content of pasture and other feeding-stuffs and their digestibility and starch equivalent. J. Agric. Sci..

[40] Kienzle E., Fehrle S., Opitz B. (2002). Interactions between the apparent energy and nutrient digestibilities of a concentrate mixture and roughages in horses. J. Nutr..

[41] Hintz H.F., Argenzio R.A., Schryver H.F. (1971). Digestion coefficients, blood glucose levels and molar percentage of volatile acids in intestinal fluid of ponies fed varying forage-grain ratios. J. Anim. Sci..

[42] Gobesso A.A.D.O., D’Auria E., Prezotto L.D., Rennó F.P. (2008). Replacement of corn by ground or extruded sorghum in diets for horses. R. Bras. Zootec..

[43] Kalantari R.K., Rouzbehan Y., Fazaeli H., Direkvandi E., Salem A.Z. (2021). The effect of three levels of concentrate and grain processing on feeding behavior, nutrient digestibility, blood metabolites and fecal pH of Turkmen horses. J. Equine Veter Sci..

[44] Peiretti P.G., Miraglia N., Bergero D. (2011). Effects of oat or corn on the horse rations digestibility. J. Food Agric. Environ..

[45] Philippeau C., Sadet-Bourgeteau S., Varloud M., Julliand V. (2015). Impact of barley form on equine total tract fibre digestibility and colonic microbiota. Animal.

[46] Miyaji M., Ueda K., Hata H., Kondo S. (2011). Effects of quality and physical form of hay on mean retention time of digesta and total tract digestibility in horses. Anim. Feed Sci. Technol..

[47] Potts L., Hinkson J., Graham B., Löest C., Turner J. (2010). Nitrogen retention and nutrient digestibility in geldings fed grass hay, alfalfa hay, or alfalfa cubes. J. Equine Veter Sci..

[48] Todd L.K., Sauer W.C., Christopherson R.J., Coleman R.J., Caine W.R. (1995). The effect of feeding different forms of alfalfa on nutrient digestibility and voluntary intake in horses. J. Anim. Physiol. Anim. Nutr..

